# The Practice Market.—V
*The previous articles in this series were published in The Hospital of March 18, March 25, April 1, and April 29.


**Published:** 1911-06-17

**Authors:** 


### THE PRACTICE MARKET.—V.*

### THE PURCHASER'S POINT OF VIEW.

In the last three articles of the present series we confined our remarks to the subjects of selling a practice and taking in a partner. We now begin to address ourselves to the purchaser of a practice or a partnership. Difficult as is the way of the vendor in the practice market, the path of the purchaser is often infinitely more hazardous and unsatisfactory. The transfer of medical openings is full of uncertainties and disappointments and disputes, and the prospective buyer especially is in need of advice and warning at every stage of the negotiations.

We have already dealt with the five ordinary methods of selling a practice or taking in a partner, and have pointed out that those which are private or semi-private are in the majority of cases the most satisfactory, although business agreements between relations or friends should be drawn up quite as strictly and as fully as those between strangers. The best partnerships and practices, like the best partners and successors, are more often obtained privately than from the books of an agency, however reputable and well established the latter may be.

To the recently qualified man, seeking to settle down in private practice, various courses are open. Unless he is in the happy position of having a family opening ready for him to drop into whenever he is ready for it, there are, roughly, four methods of entering general practice for him to consider. The first method is to "squat" behind a new brass plate and hope for the best that Fate and his friends may send him: this is outside the scope of the present articles. The second is to buy a partnership in an established firm, which is perhaps the least risky way of beginning private practice, and has various other advantages, to be mentioned later. The third method is to purchase the goodwill and professional effects of a retiring practitioner, after an introduction by him to his practice and his patients for a given length of time; and the fourth is to buy a death vacancy from the representatives of a medical man recently deceased. The first method uses up least capital, the second uses up most, while the third usually calls for a larger amount of purchase-money—other things being equal—than the fourth, because the financial risk to the buyer is supposed to be less than in taking over a death vacancy. Assistantships with a view to partnership must be included under the second heading; they are not uncommon, and are often advantageous to both parties. Partnerships with a view to early succession are usually no more than introductions posing under another name for business purposes, and therefore come under the third heading.

Before he decides to enter the practice market as a buyer the young practitioner should take careful stock of himself, his equipment, and his require-

* The previous articles in this series were published in The Hospital of March 18, March 25, April 1, and April 29.

ments, both from the professional and from the social points of view. Presumably he has already taken as many resident hospital appointments as circumstances and his tastes would permit, and has at least some slight acquaintance with private practice in the capacity of *locum tenens* or assistant. To have done a few "locums" is an invaluable experience to the man fresh from the sheltered atmosphere of hospitals; it is better not to buy one's first experience of general practice in the place where one has all at stake. If he already has some practical knowledge of work in private, but is not well up in the more important special branches of medicine or surgery, it is well for him to spend some of the time of waiting for the ideal practice in, say, the eye, or throat, or skin department of his old hospital.

Before he interviews an agent or consults his friends on the senior staff of his hospital, the intending purchaser of a practice or a share would do well to consider carefully how much of his available capital he can afford to lay out in purchase money, and how much he must leave for the initial expenses of settling down and for a reserve fund. He should also make up his mind what kind of practice, within the limits of his purse, he wishes to hear of; and in this connection he must take into account his professional qualifications and experience, his tastes, ambitions, and physique, and his matrimonial prospects, if any. He should be prepared to say at once to the agent or to his former hospital "chief," or the dean of his medical school whether he is looking for an opening in town or suburb, or for a country-town or rural practice, and how much money he is prepared to invest. The man who knows exactly what he wants, and where he wants it, and how much he will pay for the right thing, enters the practice market at a great advantage. He gets the most help from everybody, and he is usually well suited long before his less-decided competitors.

(*To be continued.*)

